# Computer-Based Interventions for Problematic Alcohol Use: a Review of Systematic Reviews

**DOI:** 10.1007/s12529-016-9601-8

**Published:** 2016-10-18

**Authors:** Christopher Sundström, Matthijs Blankers, Zarnie Khadjesari

**Affiliations:** 1 Department of Clinical Neuroscience, Karolinska Institutet, Stockholm, Sweden; 2 Trimbos institute—the Netherlands Institute of Mental Health and Addiction, Utrecht, The Netherlands; 3 Arkin Mental Health Care, Amsterdam, The Netherlands; 4 Department of Psychiatry, Academic Medical Centre, University of Amsterdam, Amsterdam, The Netherlands; 5 National Addiction Centre, Institute of Psychiatry, Psychology and Neuroscience (IoPPN), King’s College London, London, UK; 6 Centre for Implementation Science, Institute of Psychiatry, Psychology and Neuroscience (IoPPN), King’s College London, London, UK

**Keywords:** Alcohol, E-health, Computer-based intervention, Internet intervention, Systematic review, Meta-analysis

## Abstract

**Purpose:**

The aim of this review is to provide an overview of knowledge and knowledge gaps in the field of computer-based alcohol interventions by (1) collating evidence on the effectiveness of computer-based alcohol interventions in different populations and (2) exploring the impact of four specified moderators of effectiveness: therapeutic orientation, length of intervention, guidance and trial engagement.

**Methods:**

A review of systematic reviews of randomized trials reporting on effectiveness of computer-based alcohol interventions published between 2005 and 2015.

**Results:**

Fourteen reviews met the inclusion criteria. Across the included reviews, it was generally reported that computer-based alcohol interventions were effective in reducing alcohol consumption, with mostly small effect sizes. There were indications that longer, multisession interventions are more effective than shorter or single session interventions. Evidence on the association between therapeutic orientation of an intervention, guidance or trial engagement and reductions in alcohol consumption is limited, as the number of reviews addressing these themes is low. None of the included reviews addressed the association between therapeutic orientation, length of intervention or guidance and trial engagement.

**Conclusions:**

This review of systematic reviews highlights the mostly positive evidence supporting computer-based alcohol interventions as well as reveals a number of knowledge gaps that could guide future research in this field.

## Introduction

The low help-seeking rate for alcohol problems is well known, with studies pointing to around 10–15 % of people with a diagnosable alcohol problem receiving some form of treatment within the health care system [1, 2]. Although there may be several reasons for this, past research has indicated the social stigma associated with having alcohol problems a leading explanation for this discrepancy [3, 4]. For over a decade, there has been a great interest in the potential of computer-based interventions in reaching people with alcohol problems. It has been proposed that these interventions may be particularly attractive for those with alcohol problems who never seek help, due to the anonymity and 24/7 accessibility that they provide. They can also be cost-effective compared to face-to-face treatment, since no additional costs are needed once the interventions are fully developed (at least if they are fully automated), and implementation of these interventions may create wider accessibility to evidence-based interventions [5, 6].

In 2005, Kypri et al. published a paper addressing the promise and potential of computer-based interventions for alcohol problems [7]. This review concluded that the interventions seemed to have strong acceptability among patients and the public, with only a handful of effectiveness studies on computer-based alcohol interventions conducted at that time. The first systematic review focussing on treatment outcomes was published in 2008 and found the evidence with regard to effectiveness inconclusive [8]. Since then, the evidence base has expanded, with studies conducted in different countries, different contexts and different populations. In light of the emerging evidence base, several systematic reviews have attempted to combine and quantify outcomes of computer-based alcohol interventions. However, the evidence base regarding effectiveness in specific problem drinking populations (e.g. students, adults, etc.) is not yet clear.

There are also knowledge gaps in terms of identifying moderators of effectiveness. Some research has found that more extensive use of theory is associated with increased effectiveness of Internet interventions on health behaviour change [9]. Potentially linked to therapeutic orientation is intervention length, which led to a greater reduction in alcohol consumption in a systematic review of brief alcohol interventions in primary care [10]. Attention has also been given to guided Internet interventions over recent years, as these have been found to be more effective at reducing symptoms of depression than non-guided interventions in an adjoining research field [11]. Furthermore, an important methodological challenge of Internet interventions and trials in general is the impact of trial (dis-)engagement and drop-out on outcomes [12]. An overview of the extent to which these moderators of outcome have been explored in the field of computer-based alcohol interventions for problematic alcohol use is currently missing in the literature.

The aim of this paper is to summarize the evidence on computer-based alcohol interventions published over the last 10 years by narratively synthesizing the findings from systematic reviews. We will address two questions: (1) are computer-based alcohol interventions effective? and (2) what impact do therapeutic orientation, length of intervention, guidance and trial engagement have on effectiveness:?

## Methods

### Search Strategy

PubMed was searched on December 9, 2015 using the following terms for English language reviews published in the last 10 years: (alcohol OR drink*) AND (internet OR web-* OR online OR ehealth OR mhealth OR digital* OR computer*). We also filtered by study design: systematic reviews, meta-analysis review and scientific integrity review.

### Selection Criteria

Systematic reviews were included if they (1) investigated the effectiveness of computer-based or Internet-based interventions to reduce alcohol use; (2) included studies that compared the intervention to a control group; (3) included alcohol consumption or alcohol-related harm as the principal outcome and (4) included randomized trials only or mostly. Reviews that included a combination of intervention modalities, such as in-person, telephone and Internet-based interventions were excluded. Reviews of multi-dimensional interventions including alcohol (i.e. interventions on co-occurring depression and alcohol misuse) were excluded. Reviews that focussed on computer-based interventions for a range of health behaviours were included only if findings from the studies on alcohol interventions were synthesized separately. We used the following as minimum quality criteria for inclusion in our review: (1) reported inclusion/exclusion criteria, (2) conducted adequate searches and (3) synthesized the data. These criteria are used to select systematic reviews included on the Database of Abstracts of Reviews of Effects (DARE) [13]. In addition, all systematic reviews needed to either assess the quality of included studies or provide detailed information about the included studies.

### Review Screening and Data Extraction

The search strategy identified 644 references. All three authors screened titles and abstracts independently. Discrepancies were resolved through discussion. After discrepancies were resolved regarding which studies to exclude, full texts were acquired from the remaining 41 studies. All authors screened the full text of these papers, and consensus was reached about 14 papers to be included. One author (C.S.) extracted data from the included studies into a template for Table 1. Data extraction was checked for accuracy (M.B. and Z.K.).

**Table 1:** Characteristics of included systematic reviews

First author	Year	Purpose	Inclusion criteria	No of trials	Population/Setting	Control/comparator conditions	Follow up	Meta-analysis	Calculation of effect size	Moderator 1: Therapeutic Orientation	Moderator 2: Length of intervention	Moderator 3: Guidance	Moderator 4: Trial Engagement	Main findings
Bewick	2008	To review published literature on the effectiveness of web-based interventions designed to decrease consumption of alcohol and/or prevent alcohol abuse	1. Online intervention 2. Alcohol consumption as focus 3. Evaluation of intervention	5	4 Student 1 General population	1 No intervention 2 Alcohol website 2 Printed material	No mention	No	No	SBI (personalized feedback)	Includes both single-session and multiple session interventions	No mention	No mention	Heterogeneous outcome measures Small sample size at follow-up Large SD’s indicating skew Lack of pure controls Large CI’s around effect size
Carey	2009	To evaluate efficacy and moderators of computer delivered interventions to reduce alcohol use among college students	1. Electronic intervention 2. Undergraduate students 3. Behavioural Outcomes 4. Enough info to calculate effect sizes	35	Student	18 waiting list/no intervention 17 alcohol relevant content	0 to 26 weeks	Yes	Yes Short-term (≤5 weeks) and long-term (≥6 weeks) d = 0.02–0.28 on several different alcohol-related outcomes	SBI: feedback, norm comparison, alcohol education, tailored material	Includes mostly single-session interventions but some multiple session interventions Length is used as moderator in analysis	Mentions human interaction in some studies and uses this in moderator analysis	Not analysed	Interventions effective at reducing specific intervals/drink-days and max quantity, but not other quantity and frequency measures or problems (short-term). Interventions effective at reducing quantity, frequency of drinking days and problems, but not other quantity and frequency measures (long-term). Significant moderators of effect: earlier publications, fewer participants, not a commercial program, include human interaction, provide education materials, no feedback on problems.
Khadjesari	2010	To determine the effects of computer based interventions aimed at reducing alcohol consumption in adult populations	1. RCT 2. Adults drinking alcohol at any level 3. Behavioural interventions 4. Stand-alone interventions only 5. Measures change in alcohol consumption	24	18 Student 3 General population 2 Employee 1 Emergency department	22 Assessment only/waiting list 3 Active comparison	2 weeks to 12 months	Yes	Yes Grammes of ethanol: −25.9 g/week (−41 to −11) Binge frequency: −0.23 days/week (−0.47 to 0)	15 SBI (personalized feedback) 5 virtual campus 1 expectancy challenge 3 extensive behaviour change intervention	Includes mostly single-session interventions but some multiple session interventions	Not eligible	Not analysed	Interventions more effective than minimally active comparators at reducing alcohol consumed per week, and binge frequency among students. Interventions as effective as active comparator groups. Less pronounced difference among students, possibly due to baseline risk, i.e. students consumed less at baseline. Future research must use suitable measures of central tendency to summarize data
Rooke	2010	1. To quantify overall effectiveness of computer-delivered interventions for alcohol and tobacco 2. To determine whether effectiveness is associated with treatment characteristics (moderators)	1. RCT with control group 2. Computer-based 3. Substance use (only tobacco and alcohol included)	34 studies, 42 articles	28 Young adult 9 Adult 30+ 3 Adolescent 3 No info	35 attention/placebo 7 active comparison	1 to 156 weeks	Yes	Yes d = 0.20 for both alcohol and tobacco Alcohol more effective than tobacco d = 0.26 vs. d = 0.12	Monitoring, normative feedback, chat, entertainment, relapse prevention These are used as moderators	Includes mostly single-session interventions but some multiple session interventions Number of treatment sessions used as moderator	Therapist involvement used as moderator	Intention to Treat (ITT) used as moderator	Effect sizes were higher for studies with control group relative to studies in which the comparison condition was an active comparison Lower effect sizes associated with studies that include entertainment
Tait	2010	To conduct a systematic review of randomized trials of web-based interventions for problematic substance use for young adults and adolescents	1. RCT 2. No-treatment control 3. Adolescents or young adults (aged ≤25) 4. Consumption measure 5. Only fully automated interventions 6. No CDROM/stand-alone computer	14	13 student 1 employee	4 assessment only 3 No intervention 3 printed material 2 web education 2 other	30 days to 12 months	Yes	Yes Overall effect d = −0.22 Level of alcohol consumption, d = − 0.12 Binge or heavy drinking frequency, d = − 0.35 Alcohol-related soc. problems, d = − 0.57	SBI: personalized feedback, norm comparisons, alcohol education	Includes mostly single-session interventions but some multiple session interventions	Not eligible	Not analysed	Small significant effect on drinking outcomes Web interventions targeting alcohol-related problems have an effect about equivalent to brief in-person interventions Interventions to prevent the development of alcohol-related problems in those who do not currently drink appear to have minimal impact
White	2010	To review the efficacy of online interventions for alcohol problems	1. Intervention delivered via the Internet 2. Focus on moderating or stopping drinking 3. RCT 4. screening assessment or intervention	17	12 student 3 community 2 employee	10 Psycho-education 7 assessment only	1 week to 12 months	Yes, but only based on 5 studies	Yes Alcohol units/ week or month Cohens d = 0.42	SBI, information, alcohol education	Includes both single-session interventions and multiple session interventions	No mention	Retention ranged from 33.4 % to 100 %, Median reported retention in treatment condition 83.4 % at 1 month, 74.5 % at 3 months, and 74.5 % at 6 months. In controls, median retention was 80 %, 70.4 %, and 74.9 %.	Overall, online alcohol interventions appear to bring small reductions in alcohol use. However, this is based on synthesis of the results of only 5/17 included studies
Riper	2011	To assess overall effectiveness of self-help alcohol e-health interventions and to perform subgroup analyses.	1. digital self-help interventions (online or offline) 2. RCT 3. Data usable in meta-analytic procedures 4. Alcohol-drinking behaviour as primary outcome variable 5. Only results from problem drinkers 6. Adult problem drinkers (18+) 7. Control group	9 (5 single session 4 extended self-help)	General	4 Alcohol leaflet 3 Waiting list 3 Assessment only	4 weeks to 9 months	Yes	Yes Hedges g Fixed Effects =0.39 (0.29–0.50); Random Effects =0.44 (0.17–0.71)	SBI Cognitive behaviour therapy	Includes both single-session interventions and multiple session interventions Extended vs single session treatment used as moderator: Single-session Mixed Effects =0.27 (0.11–0.43) Multisession Mixed Effects =0.61 (0.33–0.90)	Not included, but its relevance is mentioned in discussion	0–42 % Loss to follow-up is discussed as a limitation	Alcohol self-help interventions are effective in general Earlier reviews showing smaller effects may be due to including studies in student populations Larger effects for multisession interventions in comparison to single session interventions
Riper	2014	To conduct a meta-analysis including both guided and unguided alcohol Internet interventions	1. RCT with control group 2. Low intensity interventions on computer or mobile device, with or without guidance 3. Alcohol consumption primary outcome measure 4. age 18 or over 5. Drinkers who exceed local guidelines for low-risk drinking	16	9 community 2 workplace 1 emergency department 1 women and infant services 1 general practice 1 military 1 alcohol website	6 Assessment only 3 Waiting list 7 Alcohol info	4 weeks to 12 months	Yes	Yes Hedges g = 0.20 (0.13–0.27), 22 g/week (2.2 drinks) less at post-intervention	7 personalized normative feedback (NF) 9 Combination of NF, Motivational Interviewing and, CBT 1 generic health education approach	Includes both single-session interventions and multiple session interventions	Yes, moderator analysis performed displaying a non significant difference: 0.23 for guided, 0.20 for unguided	Substantial drop out rate mentioned as a limitation	Supports the use of both guided and unguided interventions More studies on guided interventions are needed No significant differences between single session interventions and extended ones Studies with longer follow-up needed The brevity of intervention descriptions, intervention uptake, completion rates and heterogeneity of outcome measures and follow up measures impede the ability to generalize the efficacy
Donoghue	2014	To determine effectiveness of alcohol Internet interventions over time in nontreatment-seeking hazardous or harmful drinkers	1. RCT 3. eSBI vs control group 2. Hazardous drinking 3. At least 10 participants 4. No alcohol dependence	23	16 student 7 other	19 Assessment only 2 Alcohol leaflet 2 Other	4 weeks to 12 months	Yes, but only based on 17 studies	Yes Grammes of ethanol: - <3 months −32.74 g/wk. 3–6 months −14.91 g/wk. 6–12 months −7.46 g /wk.	Brief intervention with information and advice	Mostly single-session interventions but some multiple session interventions	No mention	Drop-out in included studies range 7.6–44.9 % The possibility of using incentives to reduce drop-out is mentioned in the discussion	Review found a significant difference between intervention and control group at less than 3 months and between 3 and 6 months follow up. However, there were no significant differences at follow ups 12 months or longer
Balhara	2014	To evaluate the available evidence for the effectiveness of web based interventions for reducing alcohol use	1.Internet intervention 2. fully automated 3. RCT 4. RCT with at least one no-treatment control group	35 studies 41 articles	21student/ school/ adolescent 14 adult	29 no-intervention 7 active control	1 week to 12 months	No	No	Mostly normative personalized feedback	Mostly single-session interventions but some multiple session interventions	Not eligible	No mention	Among adults, interventions were found to be more effective in reducing alcohol consumption than a control group only in three out of 14 studies. Among students, reduced alcohol consumption was found in 10 out of 21 studies at different time points.
Bhochiboya	2015	To review Internet interventions targeting binge drinking among students	1. Binge drinking outcome 2. Internet 3. Studies on multiple health behaviours included as well 4. Studies combining face-to-face with e-intervention included 4. Student population	14	Student	10 control groups 4 experimental groups	4 weeks to 2 years	No	No	Mostly personalized feedback. Social cognitive theory, social norms feedback theory etc. are mentioned	Mostly single-session interventions but some multiple session interventions	No mention	No mention	Periodic interventions lead to larger effects than one-time interventions. Changing perceived/subjective norms seems to be effective in reducing risky drinking behaviour The reliance on self-report is a limitation to the field because of potential social desirable answering tendencies
Dedert	2015	To characterize treatment intensity and review evidence for efficacy of e-interventions for reducing alcohol consumption and alcohol-related impairment among adults and students,	1. RCT with active or inactive controls 2. Follow-up 6 months or longer 3. Online, CD-ROM, mobile applications or IVR	28	14 adult 14 student	Student population: 8 waiting list 5 attention control 1 treatment as usual Adult population: 5 waiting list 6 attention control 3 treatment as usual	6 to 12 months	Yes	Yes Grammes of ethanol: Adults: 6 months- - 25.0 g/wk. (95 % CI **−**51.9 to 1.9) 12 months −8.6 g/wk. (95 % CI −53.7 to 36.5) Students:, 6 months **−**11.7 g/wk. (95 % CI **−**19.3 to **_**4.1) 12 months −4.7 g/wk. (95 % CI −24.5 to 15.1)	Mostly brief intervention, personalized feedback and psycho-education	Mostly single-session interventions but some multiple session interventions	Yes, the review classifies studies by level of human support	No mention	General reduction by 1 drink/week, at 6 months but no difference at 12 months, Diagnostic assessment of participants would be valuable in future research More studies on intensive interventions, preferably with some form of human interaction, are needed
Leeman	2015	To evaluate the efficacy of very brief, web-based alcohol interventions for college students	1. data collection entirely web-based 2. intervention takes no more than 15 min to complete 3. intervention concerns alcohol use in general (as opposed to event-specific drinking) 4. primary focus on alcohol consumption 5. students 6. random assignment	15	Student	9 assessment only 3 attention control 2 alternate personalized feedback 1 education	1 to 24 months	No	No	All interventions were based on personalized normative feedback, and most of these were multi- component interventions with for example behavioural techniques and facts about alcohol	All included interventions were single- session	No mention	Yes, reports on attrition bias for each individual study	Nine of the 15 RCT’s included reported an ES of at least 0.20 or greater in alcohol reduction for intervention, while 2 reported an ES in the opposite direction (control group fared better)
Dotson	2015	To summarize available research and to perform a meta-analytic review of computer-delivered stand-alone personalized normative feedback (PNF) interventions for college student drinking	1. examines a stand-alone PNF drinking intervention 2. student sample 3. includes a control condition 4. reports outcomes for drinking norms and actual drinking behaviour 5. uses a pre-post experimental design with at least 28 days between baseline and follow-up 6. provides adequate information for effect size calculation	8	Student	5 assessment only 3 attention control	4 to 20 weeks	Yes	Yes Gender neutral d = 0.291 [95 % CI, 0.16 to 0.42] Gender-specific d = 0.284 [95 % CI 0.12 to 0.45] Number of drinks: Gender neutral −3.027 [95 % CI, 2.17 to 3.88] Gender-specific −3.089 [95 % CI −0.99 to 0.59]	Personalized normative feedback	Not specified, but probably mostly single session interventions	No mention	Specifies attrition rates for each included study (4.2–21 %)	Both gender-neutral and gender-specific PNF interventions reported greater reductions when compared to controls. Results were consistent regardless of intervention setting, suggesting that computer-delivered PNF is equally effective when completed in a structured setting or when completed in a non-structured setting. Researchers should assess outcomes appropriately for the population of interest, rather than selecting a measure solely because it is a common metric.

## Results

**Fig. 1:**
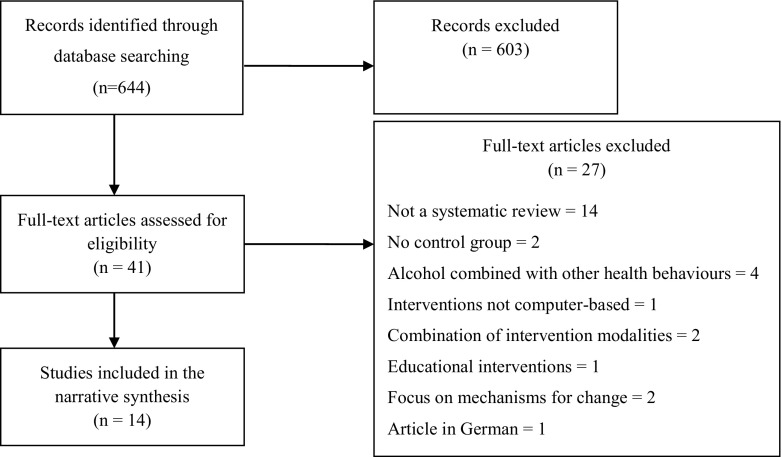
Flowchart of study selection process

Fourteen systematic reviews met the eligibility criteria (see Fig. 1). Of these, one was conducted in 2008 [8], one in 2009 [14], four in 2010 [15–18], one in 2011 [19], three in 2014 [20–22] and four in 2015 [23–26] (see Table 1). Of the included reviews, ten synthesized findings statistically with meta-analyses [14–21, 24, 26].

## Question 1: Are Computer-Based Interventions for Problematic Alcohol Use Effective?

In this section, we consider the evidence for the effectiveness of computer-based interventions and maintenance of these effects over time. We present evidence separately for reviews on mixed populations (reviews that include both adult and student populations), reviews on student populations only and reviews on adult (non-student) populations only. Seven reviews included studies from mixed populations [8, 15, 17, 18, 21, 22, 24] with two of these separately pooling findings from student and non-student populations [18, 24]. Five reviews restricted inclusion to studies conducted in student populations [14, 16, 23, 25, 26] and two reviews restricted inclusion to studies conducted in adult (non-student) populations [19, 20]. The most commonly reported outcome in the reviews was alcohol consumed within a given time frame (usually the preceding week) reported as number of standard drinking units or grammes of ethanol; some reviews also reported effects on binge drinking and alcohol-related problems.

### Reviews on Mixed Populations

Two reviews reported outcomes in standard drinking units [15, 17]; Rooke et al. 2010 reported an effect size of 0.26 representing a small effect size [15] and White et al. 2010 reported an effect size of 0.42 representing a medium effect size [17]. Two reviews reported outcomes in grammes of ethanol per week [18, 21]; Khadjesari et al. 2010 found a statistically significant reduction in the intervention group of 26 g per week (95 % CI −41 to −11), an amount reflecting about three standard drinking units (8 g of ethanol) in the UK or 2.5 glasses in European standard drinking units (10 g of ethanol) [18]. Donoghue et al. 2014, the only review to compare different follow up times in relation to outcomes, concluded that effects on alcohol consumption were sustained at 3 months, with a mean difference of −32.74 g of ethanol, (95 % CI −56.80 to −8.68), slightly smaller effects at 3 months to less than 6 months (mean difference –17.33 g, 95 % CI –31.82 to −2.84) and from 6 months to less than 12 months follow-up (mean difference –14.91 g, 95 % CI −25.56 to −4.26). No significant effects were maintained after 12 months (mean difference –7.46 g, 95 % CI −25.34 to 10.43**)** [21]. One review without meta-analyses, Balhara et al. 2014 [22], considered evidence for the effectiveness of computer-based alcohol interventions inconclusive pointing to negative findings in some RCTs.

### Reviews on Student Populations^1^

Three reviews reported outcomes in standard drinking units [14, 16, 26]. In Carey et al. 2009’s review, computer-based interventions were found to show small effects on alcohol use, ranging from 0.09 to 0.28 for all follow-up time-points [14], while Tait et al. 2010 found a minimal effect size in alcohol consumption reduction (d = 0.12, 95 % CI 0.10 to 0.34) [16]. Dotson et al. 2015 found a small effect size on consumption (d = 0.29, 95 % CI 0.16 to 0.42) and also reported a reduction of about three drinks [26]. Dedert et al. 2015, the only review reporting outcomes in grammes of ethanol per week among students, found a mean difference of −11.7 g of ethanol (95 % CI −19.3 to −4.1) per week at 6 months between computer-based interventions and control groups reflecting a reduction of about 1.5 UK standard units or one European standard unit. At 12 months, no significant difference between groups was found [24]. Three reviews presented effect sizes on binge drinking [14, 16, 24]. Carey et al. 2009 reported a non-significant reduction (at >5 weeks) with a minimal effect size (d = 0.10, 95 % CI 0.00 to 0.20, [14], Tait et al. 2010 reported a significant reduction with a small to medium effect size (d = −0.35, 95 % CI − 0.64 to −0.06) [16], and Dedert et al. 2015 found no effect [24]. Three reviews presented effect sizes on alcohol related problems [14, 16, 26]. Carey et al. 2009 reported a minimal effect size at <5 weeks (d = 0.16, 95 % CI 0.03 to 0.29) [14] in line with the findings of Dotson et al. 2015 (d = 0.157, 95 % CI 0.037 to 0.278) [26], while Tait et al. 2010 found a moderate effect size (d = −0.57, 95 % CI− 0.98 to −0.15 [16]. Narratively, Bhochhibhoya et al. 2015 supported these findings [23], whereas Bewick et al. 2008 [8] and Leeman et al. 2015 [25] highlighted mixed findings.

### Reviews on Adult (Non-Student) Populations^2^

Riper et al. conducted two reviews focusing on adult problem drinkers [19, 20]; the first of the two found a medium effect size on alcohol consumption (Hedges g = 0.44, 95 % CI 0.17 to 0.71) [19] whereas the more recently published of the two found a small significant effect size (Hedges g = 0.20, 95 % CI 0.13 to 0.27). In this later review, Riper et al. also reported a significant reduction of 2.2 “alcohol consumptions” (22 g of ethanol) per week (95 % CI 0.87 to 3.46) [20]. In the review by Dedert et al. 2015, a significant reduction of 16.7 g of ethanol was found at 6 months (95 % CI −27.6 to −5.8) with no significant difference found at 12 months [24].

Khadjesari et al. 2010 conducted a sub-group analysis of studies with student populations and found the effectiveness of computer-based interventions to be less pronounced than in the wider meta-analysis of mixed student and non-student adult populations [18].

## Question 2. What Is Known about Moderators of Effectiveness and Trial Engagement?

In this section, we present evidence about moderators of effectiveness and trial engagement. Specifically, we focus on four potential moderators: (1) therapeutic orientation, (2) length of intervention, (3) guidance and 4) trial engagement. Reviews that address these associations are presented in Table 2.

**Table 2:** Moderators of outcome and engagement addressed in the systematic reviews

Theme	Studies
1. Therapeutic orientation—outcome	Bewick 2008 [8], Carey 2009 [14]^a^, Rooke 2010 [15]^a^, Tait 2010 [16], White 2010 [17], Riper 2014 [20]^a^, Donoghue 2014 [21], Balhara 2014 [22], Bhochhibhoya 2015 [23], Dedert 2015 [24], Leeman 2015 [25]^a^.
2. Length of intervention—outcome	Carey 2009 [14], Rooke 2010 [15]^a^, White 2010 [17], Riper 2011 [19]^a^, Riper 2014 [20]^a^, Balhara 2014 [22], Bhochhibhoya 2015 [23], Dedert 2015 [24].
3. Guidance—outcome	Carey 2009 [14]^a^, Rooke 2010 [15]^a^, Riper 2011 [19], Riper 2014 [20]^a^, Dedert 2015 [24].
4. Trial engagement—outcome	White 2010 [17], Leeman 2015 [25]^a^, Dotson 2015 [26].
5. Therapeutic orientation—trial engagement	–
6. Length of intervention—trial engagement	–
7. Guidance—trial engagement	–

^a^Theme is addressed quantitatively using meta-analytic techniques.

### Therapeutic Orientation—Outcome

Eleven of the 14 reviews (79 %) addressed the association between therapeutic orientation and outcome. Four of these addressed this theme quantitatively [14, 15, 20, 25]. Carey et al. 2009 found that computer-based interventions were more successful at reducing heavy drinking frequency at long-term (≥6 weeks) when they did not provide feedback on alcohol-related problems (β = −0.63, *p* < 0.01) [14]. Rooke et al. 2010 compared interventions using normative feedback and relapse prevention with those that did not. No significant association was reported (normative feedback Q_between_(1) = 0.06, *p* = 0.80; relapse prevention Q_between_(1) = 0.01, *p* = 0.98) [15]. Riper et al. 2014 [20] performed subgroup analyses focussing on the relation between therapeutic orientation and outcome. Of the 16 studies included in the review, seven applied a single-focus therapeutic strategy, mostly personalized normative feedback, while the other nine studies used combined treatment approaches based on motivational interviewing, personalized normative feedback, cognitive-behavioural therapy and/or behavioural self-control and change principles. No significant association was found. Leeman et al. 2015 compared studies of personalized normative feedback interventions with studies of multi-component interventions and did not find any differences in effect sizes [25]. Other reviews addressed this issue narratively. The review by Bewick et al. 2008 suggested research on which elements of personalized feedback are related to outcome and whether those elements are different for low and high-risk drinkers [8]. Bhochhibhoya et al. 2015 found that in many studies, a theory-driven approach to interventions development was lacking, and of the included studies in this review, only 3 out of 14 utilized a theory-based intervention [23].

### Length of Intervention—Outcome

Eight reviews (57 %) addressed the association between length of intervention and outcome. Three of these addressed this theme quantitatively [15, 19, 20]. Rooke et al. 2010 found a non-significant association between number of sessions and treatment effect (*r* = 0.17, *p* = 0.34) [15]. Riper et al. 2011 [19] found a significant difference (*p* = 0.04) between single session interventions (g = 0.27, 95 % CI 0.11 to 0.43) and more extended Internet-based self-help interventions (g = 0.61, 95 % CI 0.33 to 0.90) in sub-group analyses. However, in a more recent review by Riper et al. 2014 [20], no significant associations were found in a meta-regression between number of sessions and effect size (b = −0.0001, 95 % CI −0.004 to 0.003). White et al. 2010 noted that the pre­post differential effect size for brief personalized feedback programs (d = 0.39) was somewhat smaller than the effect size for the multisession modularized programs (d = 0.56) [17]. Some reviews addressed the theme narratively; Dedert et al. 2015 stated that variability in treatment intensity was insufficient to formally test its association with outcomes [24], while Bhochhibhoya et al. 2015 concluded that more prolonged, multi-session interventions seem to be more effective than one-time interventions [23].

### Guidance—Outcome

The association between guidance and outcome was addressed in five reviews (36 %). In three of these, some form of quantitative analysis on this theme was performed [14, 15, 20]. Carey et al. 2009 found that computer-based interventions were more successful in reducing alcohol-related problems at short-term (≤5 weeks) when including human interaction vs. using the computer alone (β = −0.53, *p* = 0.02) [14]. Rooke et al. 2010 compared interventions with minimal therapist contact (*n* = 32), moderate therapist contact (*n* = 8) and major therapist contact (*n* = 2). No significant association was reported, Q_between_(2) = 3.29, *p* = 0.19 [15]. Riper et al. 2014 did not find an association between therapist guidance and outcome: guided (g = 0.23) and unguided (g = 0.20), *p* = 0.73 [20]. As suggested by both Dedert et al. 2015 [24] and Riper et al. 2014 the variability in amount of guidance, and the lack of published studies on guided interventions, may not yet be sufficient for a sound evaluation of its effect on alcohol-related outcomes.

### Trial Engagement—Outcome

Three reviews mentioned the possible association between trial engagement (or its reverse, drop-out) and outcome (21 %) with one of them presenting a quantitative analysis on this theme. Leeman et al. 2015 did not find differences in effect sizes between studies with retention rates at follow-up of more than 70 % versus less than 70 % [25]. White et al. 2010 reported retention in the intervention groups of the included trials ranging from 38.9 to 100 %, with a median of 74.5 % at 6 months; retention in control groups were quite similar with a range of 33.4 to 100 % and a median of 74.9 % at 6 months [17]. Dotson et al. 2015 reported drop-out rates in the included studies, ranging from 4.2 to 21 % [26]. This review in addition contended that researchers should not only measure drop-out rates but also find a way to measure whether participants actually pay attention to the intervention content as there are indications that some participants may engage in other activities while completing Internet interventions [27] in [26]].

### Moderators of Trial Engagement

None of the reviews explicitly report on the association between therapeutic orientation, length of intervention and guidance and trial engagement.

## Discussion

With few exceptions, across the systematic reviews eligible for this review, computer-based alcohol interventions are reported as being more effective in reducing alcohol consumption than control groups, albeit to different degrees. Effect sizes are mostly in the small range reflecting a weekly reduction of between two and three UK units or between one and 2.5 European units. Furthermore, effects seem to decay over time and may disappear completely after more than 12 months, although few studies include such long follow-ups. Interventions on students tend to render slightly smaller effects on alcohol consumption than interventions on adults/non-students. The impact of interventions on frequency of binge drinking and harm is not clear. Regarding moderators, there is at present no clear evidence for the superiority of one therapeutic orientation over another. There is mixed evidence of an association between length of intervention and outcome; some reviews found support for the hypothesis that the longer the length or duration of an intervention, the larger its effects, but not all. There is also mixed evidence for an added effect of guidance. Lastly, there is a lack of evidence regarding impact of trial engagement on outcome, with only one review addressing this issue quantitatively.

Despite positive findings in the included reviews, a few recent large-scale pragmatic trials on computer-based interventions have reported null findings. Kypri et al. 2014 found no significant reductions in volume or frequency of alcohol consumption in a large trial conducted in seven New Zealand universities (5135 students) [28] and another recently conducted trial in a workplace setting also found no differences (1330 employees) [29]. Whilst these studies provide different reasons for their null findings, reactivity of assessment, caused by the screening test [30] and other features of the research process [31] may provide some explanation. The findings of these trials may also be a function of their pragmatic designs, where implementation in “real world” contexts may hinder the effectiveness of brief alcohol interventions [32]. Future reviews would benefit from grading the extent to which trials measure the efficacy or effectiveness of interventions [10, 33] and exploring the extent to which this impacts on their findings.

Although the reviews found no utility of therapeutic orientation as a moderator, there was some evidence that longer interventions are more effective than briefer ones. However, it may be difficult to disentangle effects of therapeutic orientation and length of intervention in the field of computer-based interventions for problematic alcohol use. These interventions mainly fall into two therapeutic traditions: brief interventions and cognitive behaviour therapy/relapse prevention. Computer-based brief interventions are usually single-module meant to be used only once, and computer-based cognitive behaviour therapy/relapse prevention interventions usually consist of several modules intended to be used repeatedly over an extended period of time. This inter-relation of therapeutic orientation and length of intervention complicates findings. To illustrate, in a study by Cunningham 2012, two Internet-based interventions were directly compared; one brief intervention with personalized feedback intended for completion in a single session but accessible for repetition should the user wish to use it again, and one cognitive behaviour therapy/relapse prevention intervention consisting of several modules specifically intended for repeated use over an extended time period [34]. Although the extended version was shown to be more effective in reducing alcohol consumption, the data does not tell us whether therapeutic orientation or intervention length (or a combination of the two) was the (more) effective component. This considered, it is important that future analyses on computer-based interventions find ways to disentangle the relationship between these two moderators.

The potential of the Internet in delivering effective, individual-level interventions at population level is currently being realized by health agencies across Europe. The National Health Service in England offers the One You Drinks tracker app [35], the Swedish alcohol monopoly Systembolaget has developed the Promillekoll app which focuses on blood alcohol concentration [36] and the intervention database on the Dutch portal for Health Promotion and Prevention supported by the Netherlands Ministry of Health, currently lists 14 alcohol Internet interventions [37]. Whilst this is a promising development, it is important that interventions intended to be widely disseminated under-go robust evaluation and that effective implementation strategies, best suited to their context, are used [38]. Furthermore, if these interventions are intended to create a public health impact, it is vital to extend the reach of them to the whole target population. Accordingly, a relevant theme for future research would be to find effective ways to get the target population to find and access the intervention. A recent review on the possibilities of using online methods to recruit participants of Internet-based trials for a variety of health domains indicates that although online recruitment is promising for this, more empirical evidence is needed [39].

## Strengths and Limitations

A significant strength of this review is that it followed a robust methodology and answered two specific questions determined a priori, with findings synthesized narratively in response to these questions. However, some limitations should be mentioned: Whilst we had minimum quality criteria for the inclusion of systematic reviews in our review, we did not undertake a quality assessment of the reviews. For reviews published until the end of 2014, however, critical summaries are available from the Database of Abstracts of Reviews of Effects (DARE) [13]. Furthermore, discussion of moderators was not based on experimental data but was limited to meta-regression (i.e. observational data) in the included reviews. There is a growing literature that uses experimental design to explore the impact of some of these moderators [34, 40–42]. Finally, some individual RCT’s are included in several reviews; the results of the reviews are thus not fully independent of each other.

## Recommendations for Future Research

A number of themes worth considering in future research have been identified in our review of systematic reviews on computer-based alcohol interventions, which has led us to four recommendations.

Firstly, there is a clear lack of studies with long-term follow-ups. Most primary studies and (hence) reviews have follow-up durations of 6 months post-randomisation or shorter. More outcome data of 12 months post-randomisation (or longer) would allow a knowledge base with regard to long-term effects to be established. Positive findings would support the acceptance of these interventions as valuable high quality treatment. Negative findings (no or very limited maintenance of effects at 12 months post-randomisation) could guide improvements to interventions, with for example a focus on possible merits of booster sessions.

Secondly, although two out of three reviews that analysed guidance quantitatively found no effect of this moderator, there are several individual studies that have reported a clear and significant added effect of guidance [42, 43]. Given that guidance has been shown to improve effects in studies on Internet interventions in other problem areas [44], more research on the impact of (different levels of) guidance in computer-based alcohol interventions is needed to clarify what amount, if any, leads to optimal effect. Cost-effectiveness would also be an important consideration here, as therapist involvement constitutes a major cost driver [45].

Thirdly, even though there is a steady evidence base for computer-based alcohol interventions in adult and student populations, there is lack of evidence in other populations such as patients, employees and ethnic minority groups. Future primary studies and reviews should limit inclusion to these or other populations, in order to demonstrate utility of interventions in these specific groups.

A fourth and final recommendation concerns trial engagement: many reviews (and primary papers) mention the potential negative impact of trial drop-out on the quality and validity of the available literature. However, as of yet, none of the outcome-oriented reviews performs meta-regression on moderators of trial drop-out, nor on approaches to foster engagement. Addressing these issues quantitatively would be a valuable contribution to current evidence and could inform intervention developers and researchers on potential mechanisms to foster engagement, the impact of addressing these mechanisms, and means to reduce intervention and trial drop-out.

## Footnotes

1 Even though Bewick et al. 2008 and Tait et al. 2010 did not restrict inclusion to a student population, they both are included in this section since 4 out of 5 studies and 13 out of 14 studies respectively were conducted in student setting.

2 All reviews mentioned in this subsection include a small number of studies in non-health-care settings such as the workplace and the military, and trials in health care settings, such as General Practice, emergency departments, and women and infant services. These findings tend to be pooled with all non-student studies, due to their small numbers.
